# Predictors of Mortality in Posterior Circulation Ischemic Stroke

**DOI:** 10.3390/medicina62081433

**Published:** 2026-07-23

**Authors:** Sanja Rsovac Ivanović, Filip Vitošević, Damljan Bogićević, Dejana Senji, Ljubica Nikčević Krivokapić, Marjana Vukićević, Nemanja Rančić, Dragan Mašulović, Biljana Georgievski-Brkić

**Affiliations:** 1 Special Hospital for Cerebrovascular Disease “Saint Sava”, 11000 Belgrade, Serbia; filipvitosevic@gmail.com (F.V.); damljanbogicevic@yahoo.com (D.B.); ljubicanikcevic@yahoo.com (L.N.K.); brkicbiljana15@yahoo.com (B.G.-B.); 2 Clinical Hospital Center Zemun, 11000 Belgrade, Serbia; senjidejana@gmail.com; 3 Faculty of Medicine, University in Belgrade, 11000 Belgrade, Serbia; 4 Faculty of Medicine, University in Kragujevac, 34000 Kragujevac, Serbia; 5 Institute of Radiology, Military Medical Academy, 11000 Belgrade, Serbia; 6 Faculty of Medicine of the Military Medical Academy, University of Defence, 11000 Belgrade, Serbia

**Keywords:** CT perfusion, posterior circulation ischemic stroke, mortality prediction, neuroimaging, the Israeli Vertebrobasilar Stroke Scale

## Abstract

*Background and Objectives*: Posterior circulation ischemic stroke (PCIS) is a life-threatening disease with a poor prognosis. It has nonspecific symptoms. It is very difficult to diagnose. The aim of the study is to examine to what degree radiological methods, in addition to standard clinical indicators, can serve as a reliable and independent predictor of death in patients with PCIS. *Materials and Methods*: It is a retrospective study on 175 patients at the Special Hospital “Saint Sava” (2023–2025) with symptoms of acute PCIS, who did not have proven PCIS on the initial non-contrast computed tomography (NCCT). The radiology protocol included NCCT, CT perfusion (CTP) and CT angiography (CTA). The experimental group (*n* = 116, CTP+) and the control group (*n* = 59, CTP-) were monitored clinically and radiologically, with a follow-up NCCT after 24 h when all had confirmed acute PCIS. At discharge, the outcome was monitored on the National Institutes of Health Stroke Scale (NIHSS 2) and the Modified Rankin Scale (mRs). *Results*: Of 175 patients with acute PCIS, 24% died within 30 days. Those who died were older (*p* < 0.05), had a lower Posterior Circulation Alberta Stroke Program Early CT Score (pcASPECT), and more often had hyperdensity (26.2% vs. 7.5%), occlusions (69% vs. 31.6%), and symptomatic blood vessels on CTA and CTP+ (81% vs. 61.7%, *p* < 0.01). Multivariate regression singled out CTP+ (OR = 4.08), complications (OR = 2.96), Israeli Vertebrobasilar Stroke Scale (IVBSS) (OR = 1.22) and Trial of Org 10172 in Acute Stroke Treatment (TOAST) (OR = 1.92) as predictors of mortality, with shorter survival in CTP+ (949 vs. 1449 days, Log-rank *p* = 0.015), more NIHSS/mRS, lower Glasgow Coma Score, and more frequent obesity in the deceased. *Conclusions*: Negative CTP represents the protective factor against death in patients with acute PCIS. Complications, higher IVBSS, and TOAST classification (large vessels atherosclerosis) represent an increased risk. The importance of CTP for early detection of acute PCIS and early risk assessment is highlighted.

## 1. Introduction

Ischemic stroke (IS) is the third leading cause of mortality and disability globally, despite significant advances in diagnosis and treatment [1,2]. Worldwide, a large number of people (about 93.8 million) have survived IS [1,3]. IS accounts for about 80% of all strokes, and 20–25% belong to IS in the posterior cerebral circulation [4,5]. Posterior circulation ischemic stroke (PCIS) represents an already very difficult problem not only regarding treatment but also from a diagnostic perspective. It is less frequent than in the case of anterior circulation, and at the same time it presents a serious medical condition because of the poor prognosis due to the involvement of vital centers [6].

The planning and proper treatment, along with the recognition of patients with high risks of dying, are of key importance. For this reason, methods that, in addition to confirming the diagnosis, also enable the assessment of the prognosis of the disease are becoming increasingly important. IS can result from the occlusion of small blood vessels, cardioembolization due to rare or specific causes (such as arterial dissection, vasospasm, migraine, thrombophilia, vasculitis, venous thrombosis, drug and medication abuse, and others), and undetermined causes, as confirmed by the Trial of Org 10172 in Acute Stroke Treatment (TOAST) [7], which helps identify the cause of IS. Atherosclerotic stenosis and cardioembolization are the most common causes of IS [8,9]. The complicated anatomy of the posterior fossa itself, connecting the vertebrobasilar circulation with the carotid system, presents a great challenge [10,11].

The clinical challenge is establishing the diagnosis, especially because IS of the posterior basin presents with nonspecific symptoms [12] such as nausea, vomiting, dizziness and visual disturbance [5,8,10,11,13]. Recognizing the symptoms in time is crucial, but it poses significant challenges [5,14,15]. Often, the National Institutes of Health Stroke Scale (NIHSS) is inadequate for posterior basin IS. The Israeli Vertebrobasilar Stroke Scale (IVBSS) is a neurological clinical scale for more accurate assessment of neurological deficit in comparison to the NIHSS, which is not adequate enough because it does not measure visual acuity, memory impairment and Horner’s syndrome, vertical gaze palsy and nystagmus, which are frequent symptoms of acute PCIS [16,17]. A similar problem was highlighted in the study by Jones SP and associates, who found out that 2–52% of all acute PCIS were not identified by emergency services, precisely for this reason [18]. Often, patients do not show up for an examination on time. Approximately 30% of missed posterior strokes in young people who come to the emergency department are primarily because they are not suspected at first because of their age and much more often because of atypical symptoms [14].

When people have a stroke they lose a lot of brain cells fast, about 1.9 million neurons per minute in the ischemic area, so that is a reason to say “time is brain” [19,20]. This is precisely why you need to react quickly. In the first hours after symptoms appear, it is necessary to perform a non-contrast computed tomography (NCCT) of the endocranium to rule out tumors and bleeding [21,22], and then detect early signs of acute PCIS (loss of gray–white attenuation and hyperdense sign of blood vessels—“ribbon sign”) [23,24], as well as calculation of the Alberta Stroke Program Early CT Score (ASPECT) to decide on further therapy, i.e., Posterior Circulation Alberta Stroke Program Early CT Score (pc—ASPECT) modified for the posterior circulation [25]. Since the changes are visible only after a few hours, NCCT is not sensitive enough for early recognition of acute PCIS [26,27], but, on the other hand, it is cost-effective and available and allows rapid image acquisition [28]. Studies indicate that the sensitivity of computed tomography perfusion (CTP) in identifying PCIS is much higher [29], as much as 72%, compared to only 25% for NCCT of the endocranium [30]. Some authors believe that the sensitivity is around 40% [31]. CTP represents a modern radiological method that allows the measurement of blood flow in the brain with the application of a contrast agent, that is, a technique that provides a dynamic view of brain perfusion, with the possibility of distinguishing irreversible infarction (core) from reversible ischemic tissues (penumbra) [32,33,34]. This method allows for recording in less than 60 s, which is crucial given the time-sensitive nature of the procedure [27,32,33,35].

Based on CTP, we can determine how reversible the part of the brain that we can still react to is to decide which type of therapy to choose and to assess whether there is a risk of complications such as hemorrhagic transformation [32]. The limitations of CTP are difficulties in interpretation due to artifacts from the bones of the posterior cranial fossa [27,36], the complexity of the anatomy [12], and cardiac dysfunction [37].

After CTP, patients get computed tomography angiography (CTA). The “one step” approach implies protocols that combine NCCT, CTA and CTP into a single view. Clinical research proves that the use of CTP improves the recognition and selection of patients for emergency therapy and the recognition of patients with a high risk of death [32], as well as a more rational use of intensive care unit resources.

The aim of the study is to examine to what degree radiological methods, in addition to standard clinical indicators, can serve as a reliable and independent predictor of death in patients with PCIS and thus contribute to the formation of clear and specific guidelines for the high-risk population and personalized therapy.

## 2. Materials and Methods

We conducted a retrospective study at the “Saint Sava” Special Hospital for Cerebrovascular Diseases in Belgrade, Serbia. The research included 175 patients who were hospitalized in the period from January 2023 to September 2025. The study included patients of both sexes, aged 18 to 90 years, who met the requirements of the PCIS. The experimental group consisted of 116 patients who had perfusion deficit on CTP, and the control group consisted of 59 patients without perfusion deficit on CTP. All patients had developed acute PCIS after 24 h on NCCT of the endocranium.

The study included patients who had acute PCIS manifested in the first 24 h and did not have proven acute IS on the initial NCCT of the endocranium. On admission to the department, all patients were followed up clinically, neurologically, and neuroradiologically. An adequate medical history was obtained for each patient, including key data such as the time of occurrence of complaints and the type of symptoms; internal and neurological examinations (IVBSS and NIHSS) were performed upon admission and discharge, along with the Glasgow Coma Scale (GCS) and the Modified Rankin Scale (mRS), followed by appropriate laboratory analyses.

The neuroradiological protocol included NCCT of the endocranium, CTP and CTA. An initial NCCT of the endocranium (General Electric Revolution 128 (120 kV, 200 mA, slice thickness 110 mm), GE HealthCare, Chicago, IL, USA) excluded the presence of hemorrhage and distinguished acute IS, and also showed early signs of acute IS (loss of gray–white attenuation and hyperdense sign of blood vessels—“ribbon sign”). In each patient, an ASPECT score and Fazekas score to assess the percentage of white matter were calculated. Patients who had already proven IS on NCCT of the endocranium, regardless of the time of presentation to the hospital, were not considered. After the patients signed the consent for the application of the contrast medium, CTP was performed on all of them (128 MSCT machine, manufacturer: General Electric Medical systems Revolution, Chicago, IL, USA, 80 kV, 170 mA, field width 110 mm, gantry tilt 0, rotation time, FOV, matrix, 40 mL of contrast medium, GE HealthCare). Following administration of the contrast agent and data acquisition, the imaging session will be transferred to the work station as the analysis begins (software—GE Advantage Server 3.2 ext. 4.6). It is necessary to determine the arterial input functions (AIFs) and venous output functions (VOFs) shown by the two curves. The VOF peak is easier to detect than the AIF peak because the blood vessel’s larger caliber, particularly in the posterior fossa [27,38], such as the superior sagittal sinus, facilitates this detection; however, if artifacts are present, they can be identified manually [37]. The maximum field coverage is determined by the width of the scanner’s detector array. Newer devices include almost the entire coverage of the brain and evaluate more vascular territories within the same examination and have better resolution and faster imaging [39,40].

By post-processing we obtain the following parameters: perfusion maps (cerebral blood flow—CBF, cerebral blood volume—CBV, mean transit time—MTT, time to maximum residue function—Tmax) expressed in mm^3^ [32,41] and volumetric measurement of the ischemic core and critical hypoperfusion, i.e., we can determine whether there is a significant amount of tissue that, with timely reperfusion, can be saved [27,32], and all this is visually displayed through four colored maps [42]. Each map is displayed by post-processing with a color scale where the colors correspond directly to the degree of perfusion abnormality—green indicates a normal state, yellow borderline, and red and blue critical abnormalities.

CBF is the amount of blood flowing through a certain volume of brain tissue in a unit of time (mL/100 g/min) [26,32,36,43] and green color shows normal values (50–70 mL/100 g/min), blue moderate reduction (30–50%), while red color signals critical hypoperfusion (<30% of the contralateral hemisphere), i.e., core (Table 1) [44].

CBV is the total volume of blood in a unit of brain tissue (mL/100 g) [26,32,36,43]. Green color shows normal values from 2 to 4 mL/100 g, yellow–green shows borderline values (1.5–2 mL/100 g), while blue indicates a critically low volume that is less than 2 mL/100 g, i.e., core (Table 1) [41].

MTT is the average time of blood transit through the cerebral vascular network expressed in seconds (s) [26,32,36,43]. Green color on MTT indicates normal values that are less than 6 s. Blue color shows extended time (6–12 s). If the time is longer than 12 s and shown in red, it is considered significant hypoperfusion (>12 s) (Table 1) [44]. MTT is automatically calculated by the formula CBV/CBF = MTT [26,36].

The Tmax map is the most sensitive and represents the delay time in seconds until the contrast reaches its maximum concentration in the tissue. Time less than 4 s and shown in green indicates normal time (<4 s), light blue slightly extended (4–6 s), yellow moderate delay (6–8 s), and orange significant delay (8–10 s). If the delay is greater than 10 s and displayed in red, it indicates an extreme delay (>10 s) (Table 1). Patients for recanalization therapy are determined by a large red–yellow Tmax zone that significantly exceeds the blue CBV zone [44].

If the volume of the CBF map is greater than the CBV map, it means that there is a penumbra zone (Table 2) [32]. In case the territory of the infarcted field coincides with the perfusion defect, there is no need for treatment because there is no tissue to protect [22,26].

**Figure 1:**
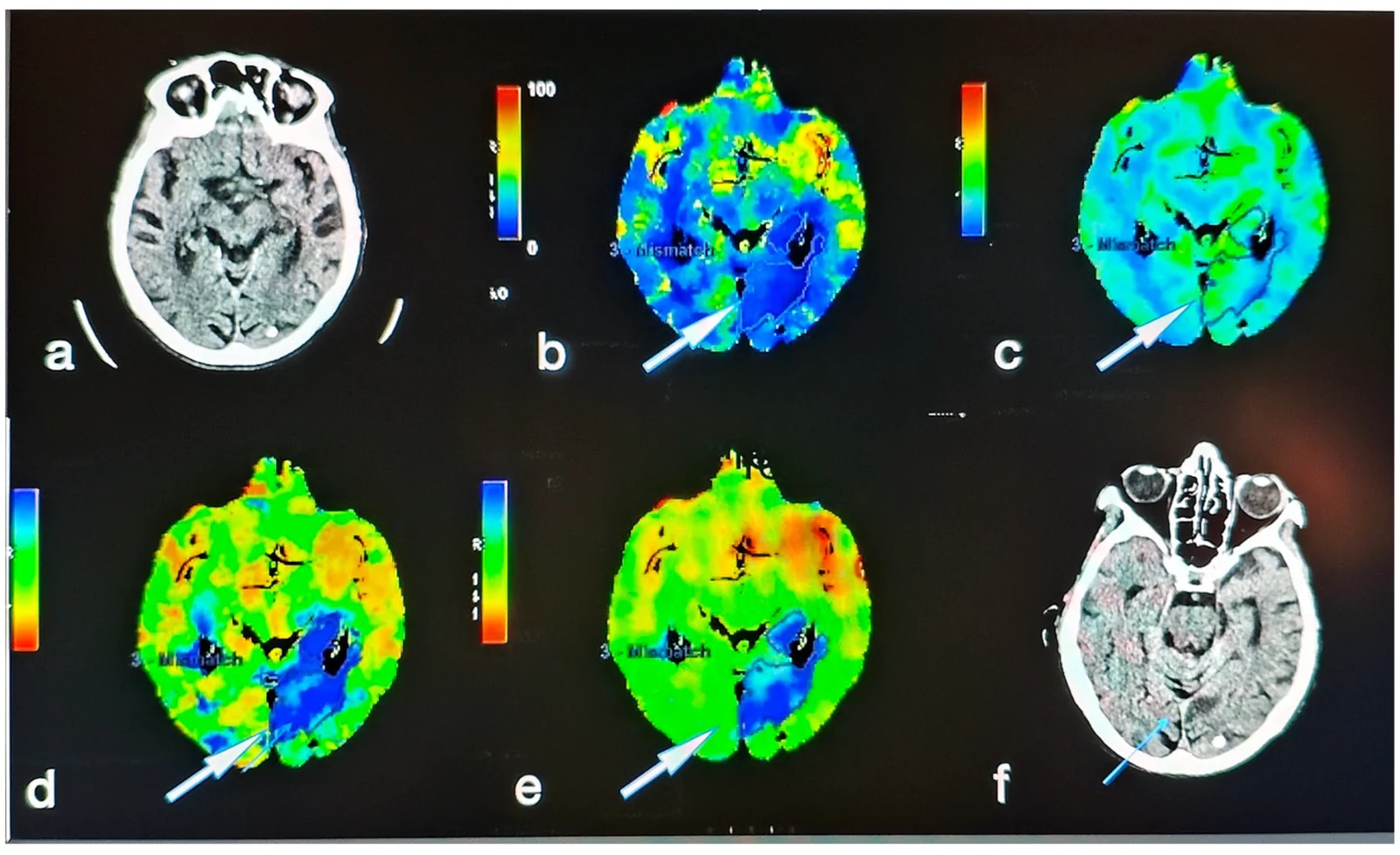
An 82-year-old female patient from Hospital “Saint Sava”, with instability and right-sided weakness. NCCT without pathological changes, ASPECT 10 (**a**). CTP showing a perfusion deficit in the ACP irrigation zone on the left at CBF (**b**) and CBV (**c**), with prolonged MTT (**d**) and Tmax (**e**) and a penumbra zone of 21.12 mL (white arrow). Follow-up NCCT after 24 h shows an acute ischemic lesion on the left occipital lobe (blue arrow) (**f**).

**Figure 2:**
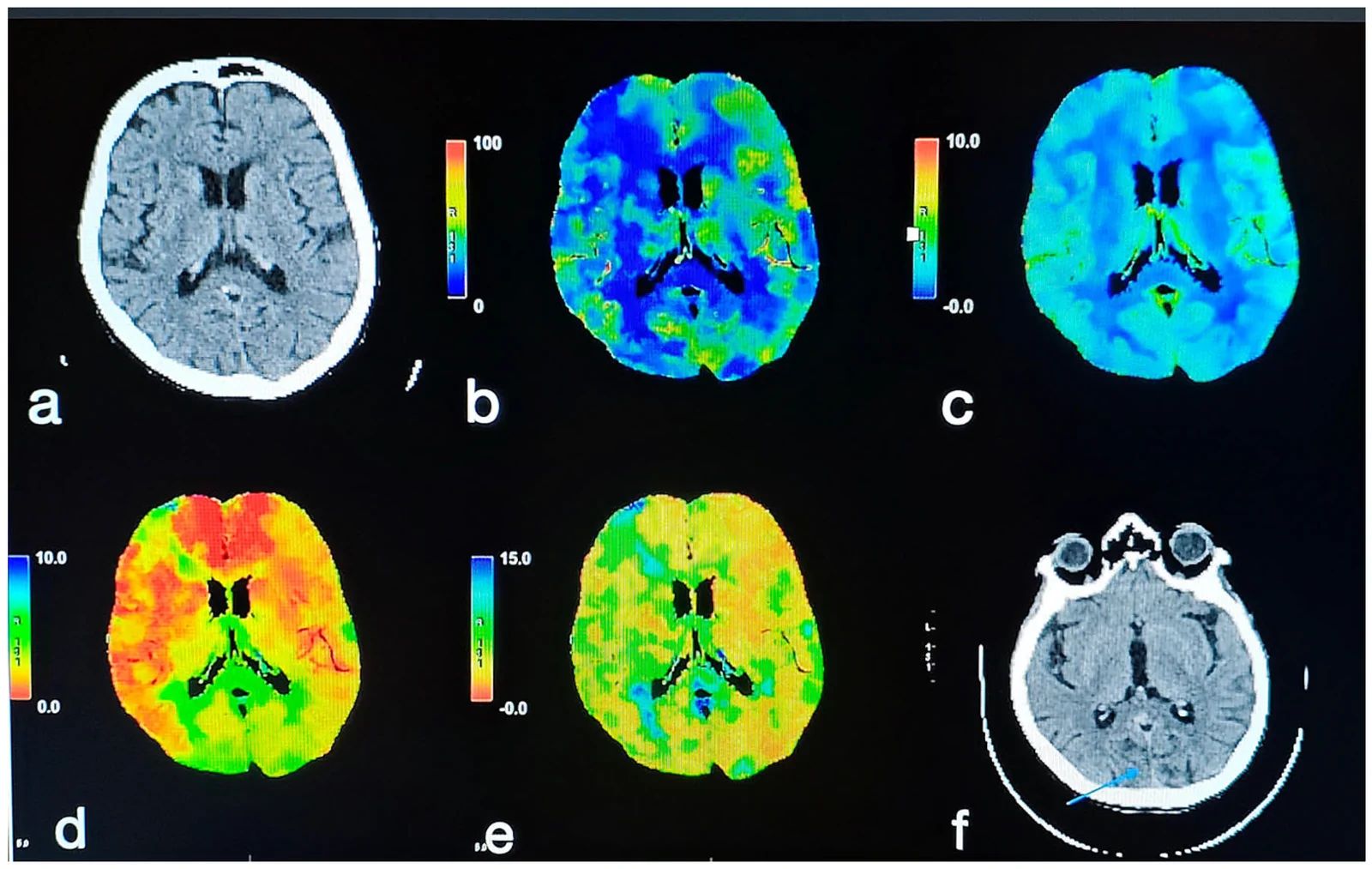
An 83-year-old female patient from Hospital “Saint Sava”, with dizziness and right-sided weakness. NCCT without pathological changes, ASPECT 10 (**a**). CTP representation showing CBF (**b**), CBV (**c**), MTT (**d**) and Tmax (**e**) without perfusion deficit. Follow-up NCCT after 24 h shows an acute ischemic lesion on the left occipital lobe (blue arrow) (**f**).

The maps were interpreted by an experienced radiologist. In the experimental group, there were numerical values (Figure 1), and in the control group, the perfusion maps were without perfusion deficit (Figure 2).

In the same act, a CTA was also performed on all patients, which showed possible obstructions as well as determined the type of blood vessels. Patients underwent a control NCCT of the endocranium 24 h after arrival at the hospital to prove acute IS and rule out complications, and in case of deterioration of the patient’s condition, earlier. Based on the obtained analyses, which include radiological results, the time of onset of symptoms, and the clinical picture, the team made a decision regarding the therapeutic procedure in collaboration with the interventional neuroradiologist.

At the time of discharge, all patients were diagnosed with the underlying cause or etiology of their acute stroke according to TOAST criteria. Based on the information from the CTA analysis, it was determined which artery the patient had a problem with. Atherosclerosis of large blood vessels (LAA) was classified in the TOAST 1 category. The TOAST 2 group was determined by the internist and data on cardioembolization (CE). TOAST 3 (Occlusion of small blood vessels—Lacune), 4 (Stroke of other determined etiology—OTH) and TOAST 5 (Stroke of undetermined etiology—UND) required further diagnostic tests.

At discharge, patient outcome was monitored based on the NIHSS score (NIHSS 2) and mRs. For patients that died, a NIHSS score of 42 was taken, which is the maximum score indicating the worst possible neurological outcome. This approach was applied to ensure uniform outcome assessment across all patients; however, it should be interpreted as a methodological convention rather than a directly observed discharge NIHSS value.

All statistical analyses were performed using the Statistical Package for Social Science (SPSS software package, version 26.0; SPSS Inc., Chicago, IL, USA). Mean and standard deviation or median and interquartile range were used for the description of parametric and nonparametric numerical data, respectively. Categorical data were expressed as frequencies or percentages. Numerical continuous variables were tested with the Kolmogorov–Smirnov test to determine if their distribution was normal. Parametric data were assessed using an Independent Samples test. The Mann–Whitney test was performed to analyze the non-parametric data. A Chi-square test was used to compare categorical variables between the two groups. The univariate and multivariate logistic regression model was used to determine predictors of mortality risk. Spearman’s rank correlation was also used to analyze the association of the variables of interest. Survival analysis was also performed with the Kaplan–Meier analysis and the Log-rank test was used. Statistical significance was set at 5% (*p* < 0.05).

## 3. Results

All patients in this study were divided into those who survived 30 days after the stroke and those who died during those 30 days; that is, 24% of the total of 175 patients died. If we look at the basic characteristics of the subjects, we see that the patients who died were statistically significantly older, on average about 5.5 years (Table 3). In other characteristics, no significant difference was found between these two groups of patients.

On the other hand, when their radiological characteristics are compared (Table 4), it is noted that hyperdensity of the blood vessels on CTA was significantly more often registered in deceased patients compared to survivors (26.2% vs. 7.5%). The ASPECT score was statistically significantly lower in those who died. In those who died, occlusion was more commonly seen on CTA (69%), while in survivors, significantly more often normal findings were seen (35.2%), while occlusion was seen in only 31.6% of patients. Symptomatic blood vessels were more present in patients who died, while asymptomatic blood vessels were more common in the patients who survived. When looking at vascular lesions according to Fazekas, patients who died were most often in category 1 (69%), whereas among healthy patients, categories 0 and 1 were equally common (48% and 45.1%). CTP was also positive in those who died more often (81% vs. 61.7%).

Looking at detailed clinical characteristics in relation to survival (Table 5), those who died were statistically significantly more likely to be obese (18.8% vs. 6.1%) and more likely to have post-stroke complications (16.7% vs. 8.3%). NIHSS 1 and 2 scores were significantly higher in those patients who died, while the modified Ranking score was significantly higher. Although the GCS on admission was statistically significantly lower in those who died, the differences in the clinical sense are small (13 vs. 15). IVBSS was also significantly higher in those who died, so only 9.5% of deceased patients had a score of less than 4 compared to 29.3% of surviving patients.

Mortality after stroke has a strong positive correlation with age, so death occurs more often in older patients (Table 6). We can also distinguish a significant strong association between the death outcome and positive CTP (Table 7). A strong correlation was also found with symptomatic blood vessels, as well as with Fazekas lesions.

Clinical features of interest that positively correlated strongly with the outcome of the disease, i.e., with the occurrence of death, were the presence of obesity, higher NIHSS 1, higher NIHSS 2, higher mRs, lower GCS, lower TOAST, and higher IVBSS (Table 8).

If the prediction of the death outcome is made with the help of binary logistic regression, first by univariate and then by multivariate analysis (Table 9 and Table 10), we see that CTP, occurrence of complications, TOAST, and IVBSS score stand out as significant predictors. A positive finding on CTP increased the chance of death by 4.08 times, complications by 2.96 times, IVBSS by 1.22 times, and TOAST by 1.92 times.

**Figure 3:**
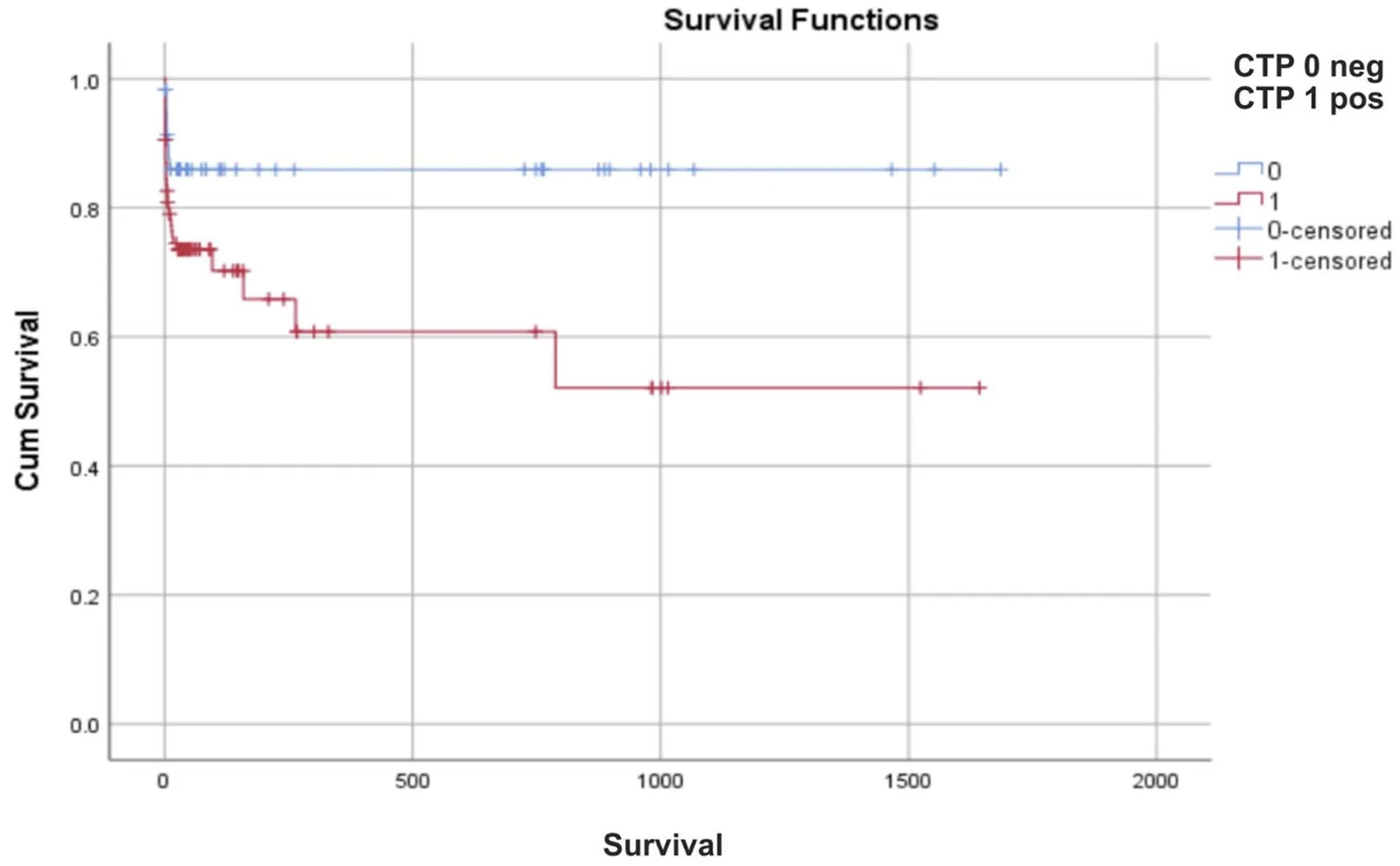
Kaplan–Meier analysis of overall survival in relation to CTP.

When the survival analysis (Figure 3) is performed in relation to CTP, as the most significant predictor of mortality, we see that the average survival in days after stroke was significantly shorter in the deceased with 949.30 (95% CI 702.55–1196.06) days compared to the survivors with 1449 (95% CI 1297.01–1601.41) days, i.e., the Log-rank Mantel–Cox test was statistically significant, *p* = 0.015. Figure 3 shows that death in patients with a positive CTP occurred in 34 (29.31%) out of a total of 116 patients, while in patients with a negative CTP result it occurred in eight (13.56%) out of a total of 59 patients.

In the CTP-positive group, there were four additional deaths after the first 30 days, while in the CTP-negative group the number of deaths remained unchanged after day 30 (Table 11).

## 4. Discussion

In this study, patients were separated into two groups: those who survived 30 days after IS and those who died within the same period. The total number of patients was 175, and the mortality rate was 24%. There was a highly statistically significant relationship between death and positive CTP.

Using binary logistic regression, first through univariate analysis and then through multivariate analysis, we identified significant predictors. These are CTP, TOAST classification, presence of complications, and IVBSS.

### 4.1. Predictive Role of CT Perfusion in Mortality

In our study, the mortality rate with a positive CTP was almost 30% (34 patients) out of a total of 116 patients, while in the case of negative CTP patients, only eight patients (13.56%) out of a total of 59 died. The overall mortality rate, in the study conducted by Furlanis et al., was 12.1% [41].

CTP has a significant positive predictive value for death after IS in both univariable and multivariable logistic regression. A negative finding on CTP was shown to be a strong protective factor against death, i.e., it indicates that patients without demonstrated perfusion abnormalities, even in the presence of clinical symptoms of IS, have a significantly lower risk of mortality compared to those with a positive finding and actually represents a protective role.

The multivariate analysis verified the prognostic value of CTP, which implies that negative CTP remains the predictor of survival, along with complications, TOAST classification, and IVBSS. This confirms that the negative CTP is actually a protective factor. A negative CTP result indicates a low-risk group of patients who have better survival rates and relatively good health, along with a need for less invasive treatment and reasonable monitoring; conversely, a positive CTP result signifies the necessity for more aggressive interventions. Our results show that a positive CTP test increases the chance of death by as much as 4.08 times. Therefore, if the CTP findings are taken into account in combination with clinical neurological scores and laboratory tests, it becomes possible to develop a risk model that will be able to discriminate between high-risk and low-risk patients. This approach allows for the adoption of a personalized treatment and rehabilitation strategy in each case.

Pallesen et al. concluded that patients with hypoperfusion on CTP had a significantly worse 12-month survival [45], which is consistent with our results. Lou C. also demonstrates in his work from 2025 that there is a strong correlation between Critical Area Perfusion Score (CAPS) and low probability of favorable outcomes in basilar artery occlusion, which is proof that CTP is associated with poor outcomes in acute PCIS [46].

Our overall mortality analysis revealed a distinct separation of the two groups over time. In CTP-positive individuals, the number of deaths rose from 30 at day 30 to 34 at day 1500, revealing that the majority of deaths happened during the first 30 days. For the CTP-negative group, there were no more deaths after day 30. Thus, a positive result of CTP is correlated with increased mortality and unfavorable survival rate. The first 30 days seem to be a crucial time period for survival. Our results are consistent with other studies showing that CTP findings in PCIS may have prognostic significance [45].

We can state that those who died following a stroke had a shorter average survival time than those who survived; specifically, the average survival time in days after a stroke was 949.30 days for those who died, compared to 1449 days for survivors, suggesting that a negative CTP result may be associated with better survival. The usefulness of CTP in practical work lies in optimal candidate choice for reperfusion treatment.

However good it is, CTP has certain limitations in the posterior fossa such as beam-hardening artifacts, limited spatial resolution, variability among post-processing software packages, and reduced sensitivity for lacunar infarctions, nearby bones which can lead to inaccurate CTP maps and misinterpretations, but we hope that newer software algorithms can help minimize these artifacts.

### 4.2. Importance of TOAST in Our Multivariate Analysis

The TOAST classification, regarding etiology, turned out to be the independent predictor of death [47]. Our analysis revealed that 31 patients with LAA died, while only 27f other patients survived. That is consistent with the pathophysiology of acute IS due to blood vessel occlusion and infarction development [48]. In case of cardioembolization, 3 patients suffered a fatal result, whereas 36 were still alive. Additionally, the death rate in 3 patients was connected with small vessel disease. Moreover, it should be pointed out that only 52% of patients were less likely to die in comparison with those who have small-vessel disease versus large-vessel disease. It was concluded that the TOAST classification, i.e., its subtypes, increases the chance of mortality 1.92 times.

A prospective study conducted by Karvelas et al. for 10 years among 1247 participants showed that there was a doubling of mortality, complications, and recurrence of stroke in TOAST classification in cases where LAA was present [49], which is similar to the results of our research. The prospective study carried out by Karvelas et al. for 10 years involving 1247 patients found out that there was a twofold increase in mortality rate, side effects, and stroke recurrence in the TOAST classification where LAA was involved [49], which is consistent with our findings.

The increased mortality rate in cardioembolization was reflected by Petty et al. in their research, which accounted for approximately 80% [50]. Also, Mehndiratta et al. indicate the importance of the death outcome in atherosclerosis of large blood vessels which is in accordance with our results, and cardioembolization [51], which is not in accordance with our results. Also, Bustamante et al. believe that survival rates are higher in patients with large vessel disease, which is not in accordance with our findings [52]. The greater factor compared to mortality and disability caused by unknown reasons was reported by D’Anna et al. [53], while other researchers did not find any significant differences [54].

### 4.3. Complications—The Strongest Risk

In our study, complications increase the risk almost threefold (OR = 2.964), i.e., they are strong predictors of mortality. The results show that complications were present in 17% of those who died versus 8% of those who survived. There is also an 86% lower probability of death if there are no complications. NIHSS scores were higher among patients who experienced complications.

Cerebral edema, entrapment, hemorrhagic transformation, and hydrocephalus are considered the most widespread. In our study, the displacement of the mediosagittal line was most frequent in 9.5% of the deceased versus 1.5% of the survivors. Second by frequency is the hemorrhagic transformation, occurring in 7% of deaths and 7% of surviving participants. Demitras et al. found that hemorrhagic transformation is a frequent complication associated with increased mortality and morbidity [55], which is consistent with our results. Bustamante et al. point out that brain edema and respiratory infections are the main post-infarction complications, especially if they occur in patients with high NIHSS above 10. In addition, hemorrhagic transformations are less likely to develop after treatment and cardiac complications [52].

### 4.4. IVBSS—Specific for Posterior Circulation

Many studies support the increased NIHSS and a lower survival rate [56]. Many studies show that the purpose of the IVBSS is to better assess the neurologic deficit compared to the NIHSS stroke scale in the case of PCIS.

However, despite the statements of many authors regarding the necessity of using NIHSS to evaluate ischemic stroke patients, IVBSS in our work turned out to be much more important. The results obtained allow us to claim that IVBSS is the parameter of poor outcome and is more significant than NIHSS. The IVBSS score was 6 in the group of living individuals and 10.5 in the deceased group.

About 29% of surviving patients had an IVBSS of less than 4, and 9.5% of those who died. In univariable analysis, an IVBSS score of less than 4 is associated with a protective effect (OR = 0.251), while each additional point on the IVBSS score increases the probability of death (OR = 1.217 per point). We conclude that elevated IVBSS values are predictors of poor prognosis.

Research shows that Furlanis et al. arrived at the conclusion that increased IVBSS values significantly correlated with poor outcomes, similar to our results, indicating the importance of more precise patient selection for endovascular therapy based on these parameters [41].

The limitations of this research consist of the small sample size, retrospective case–control study, and limited generalizability since this is a highly selected group of patients with no definite ischemic changes at initial non-contrast CT who subsequently underwent CTP and CTA according to our institutional protocol. Also, differences in imaging technique and management between centers can influence the external validity. Some important clinical parameters were not taken into account during multivariate analysis and need further evaluation in order to eliminate residual confounding factors. Future prospective multicenter studies will confirm these results.

## 5. Conclusions

A negative CTP is the protective factor against death in patients with acute PCIS, while complications and higher IVBSS and TOAST classification (atherosclerosis of large blood vessels) represent an increased risk. This highlights the importance of CTP when compared with other parameters such as NIHSS and ASPECT, encouraging its application in daily clinical practice for early detection of acute PCIS, early risk assessment, and optimization of therapeutic options. IVBSS and TOAST classification have a significant prognostic role in acute PCIS. The first 30 days seem to be a key time frame, as most deaths happen during this period. These findings also show the possible benefits of using multimodal CT, especially CTP, for early risk assessment and clinical decision making in patients with PCIS.

## Figures and Tables

**Table 1:** How to visually identify penumbra versus core.

	CBF	CBV	MTT	Tmax
Core	Red zone (<30%)	Blue zone (<2 mL/100 g)	Red zone (>10 s)	Red zone (>10 s)
Penumbra	Blue zone	Small green zone	Blue zone (6–12 s)	Blue zone

**Table 2:** Presentation of the infarct-free zone, hypoperfusion zone and irreversible zone on the maps.

	CBF	CBV	MTT	Tmax
Infarction-free zone	normal	normal	normal	normal
Hypoperfusion zone	↓	↓	↑	↑
Irreversible zone	↓↓↓	↓↓↓	↑↑↑	↑↑

The arrows indicate the severity of perfusion changes, where a greater number of arrows denotes a more pronounced abnormality.

**Table 3:** Sociodemographic and clinical characteristics of the subjects in relation to the outcome of the disease.

	Survivors *n* = 133 (76%)	Died *n* = 42 (24%)	*p*-Value
Gender:			
Male	100 (75.2%)	25 (59.5%)	0.078 *
Female	33 (24.8%)	17 (40.5%)	
Age, years	67.81 ± 12.50	7.33 ± 10.87	0.011 #
Time from complaints to examination; minutes	247 (120–605)	300 (127.5–495)	0.795 “
Time longer than 6 h	55 (41.4%)	18 (42.9%)	1.000 *
Time longer than 9 h	37 (27.8%)	9 (21.4%)	0.536 *

*—Chi-square test; #—Independent Samples test; “—Mann–Whitney test; Values are presented as number (percentage), mean ± standard deviation, or median with interquartile range (25–75th percentile).

**Table 4:** Radiological characteristics of the subjects in relation to the outcome of the disease.

	Survivors	Died	*p*-Value
Hyperdensity	10 (7.5%)	11 (26.2)	0.003 *
ASPECT score	9.64 ± 0.71	9.36 ± 0.98	0.044 #
ASPECT score < 8	14 (10.5%)	8 (19.0%)	0.236 *
Perfusion data:			
Penumbra	/	5.92 (2.50–24.00)	/
Hypoperfusion	/	6.66 (2.51–25.34)	
CORE	/	0.00 (0.00–1.88)	
Mismatch	/	8.42 (3.71–24.94)	
Territory:			
PICA	11 (8.3%)	2 (4.8%)	0.003 *
AICA	1 (0.8%)	/	
ACS	3 (2.3%)	2 (4.8%)	
ACP	65 (48.9%)	17 (40.5%)	
AB perforant branches	36 (27.1%)	4 (9.5%)	
AB main stem	8 (6.0%)	12 (28.6%)	
More territories	7 (5.3%)	5 (11.9%)	
AV	2 (1.5%)	1 (2.4%)	
Type of blood vessel:			
Normal CTA	47 (35.3%)	2 (4.8%)	<0.001 *
Significant stenosis	17 (12.8%)	3 (7.1%)	
Occlusion	42 (31.6%)	29 (69.0%)	
Dissection	6 (4.5%)	5 (7.6%)	
Insignificant stenosis	21 (15.8%)	3 (7.1%)	
Blood vessel:			
Symptomatic	53 (39.8%)	32 (76.2%)	<0.001 *
Asymptomatic	80 (60.2%)	10 (23.8%)	
Fazekas score:			
0	64 (48.1%)	5 (11.9%)	<0.001 *
1	60 (45.1%)	29 (69.0%)	
2	8 (6.0%)	7 (16.7%)	
3	1 (0.8%)	1 (2.4%)	
Positive control NCCT	133 (100%)	42 (100%)	/
CTP	82 (61.7%)	34 (81.0%)	0.034 *

*—Chi-square test; #—Independent Samples test; Values are presented as number (percentage), or mean ± standard deviation.

**Table 5:** Clinical characteristics of subjects in relation to disease outcome.

	Survivors	Died	*p*-Value
Hypertension	102 (76.7%)	37 (88.1%)	0.169 *
Atrial fibrillation	26 (19.5%)	10 (23.8%)	0.706 *
Diabetes mellitus	35 (26.3%)	16 (38.1%)	0.204 *
Blood lipids	57 (44.5%)	13 (40.6%)	0.842 *
Smoking	30 (23.3%)	6 (22.2%)	1.000 *
Alcoholism	7 (5.4%)	3 (11.1%)	0.506 *
Obesity	8 (6.1%)	6 (18.8%)	0.053 *
Therapy:			
Thrombolysis	54 (40.6%)	14 (33.3%)	0.117 *
Thrombectomy	17 (12.8%)	11 (26.2%)	
Not performed	62 (46.6%)	17 (40.5%)	
Complications:			
No complications	122 (91.7%)	35 (83.3%)	0.044 *
Hemorrhagic transformation	9 (6.8%)	3 (7.1%)	
The displacement of the mediosagittal line	2 (1.5%)	4 (9.5%)	
Hydrocephalus	/	/	
Herniation	/	/	
NIHSS 1	6.00 (4.00–8.00)	10.00 (6.75–19.25)	<0.001 “
NIHSS 1 < 5	64 (48.1%)	6 (14.3%)	<0.001 *
NIHSS 2	4.00 (2.00–6.00)	42.00 (42.00–42.00)	<0.001 “
NIHSS 2 < 5	88 (66.2%)	/	<0.001 *
mRs	1.00 (1.00–3.00)	6.00 (6.00–6.00)	<0.001 “
GCS	15.00 (15.00–15.00)	13.00 (8.75–15.00)	<0.001 “
Anticoagulant/Antiaggregant therapy	33 (24.8%)	13 (31.0%)	0.557 *
Previous ischemic stroke	13 (9.8%)	3 (7.1%)	0.835 *
TOAST:			
LAA	27 (20.3%)	31 (73.8%)	<0.001 “
CE	36 (27.1%)	3 (7.1%)	
Lacune	24 (18.0%)	3 (7.1%)	
OTH	/	/	
UND	46 (34.6%)	5 (11.9%)	
Vertigo	16 (12.0%)	4 (9.5%)	0.867 *
Nausea/Vomiting	14 (10.5%)	2 (4.8%)	0.411 *
Diplopia	11 (8.3%)	/	0.119 *
Nystagmus	/	/	
Instability	26 (19.5%)	11 (26.2%)	0.483 *
Dysphagia	/	1 (2.4%)	0.542 *
IVBSS	6.00 (4.00–8.50)	10.50 (7.00–20.25)	<0.001 “
IVBSS < 4	39 (29.3%)	4 (9.5%)	0.027 *

*—Chi-square test; “—Mann–Whitney test; Values are presented as number (percentage) or median with interquartile range (25th–75th percentile).

**Table 6:** Correlation of demographic characteristics with disease outcome—Spearman’s rho.

		Gender	Age	Time from Symptoms to Examination	Time > 6	Time > 9
Disease outcome	r	0.148	0.205	0.02	0.013	−0.062
	*p*	0.05	0.006	0.795	0.864	0.415

**Table 7:** Correlation of radiological characteristics with disease outcome—Spearman’s rho.

		Hyperdensity	ASPECT Score	ASPECT Score < 8	Territory	Type of Blood Vessel	Blood Vessel: Symptomatic/Asymptomatic	Fazekas Score	CTP
Disease outcome	r	−0.245	−0.14	−0.11	0.181	0.222	−0.311	0.331	0.174
	*p*	0.001	0.064	0.148	0.017	0.003	<0.001	<0.001	0.021

**Table 8:** Correlation of clinical characteristics with disease outcome—Spearman’s rho.

		Disease Outcome
Hypertension	r	−0.12
	*p*	0.112
Atrial fibrillation	r	−0.045
	*p*	0.554
Diabetes mellitus	r	−0.111
	*p*	0.145
Blood lipids	r	0.031
	*p*	0.693
Smoking	r	0.009
	*p*	0.908
Alcoholism	r	−0.088
	*p*	0.276
Obesity	r	−0.179
	*p*	0.022
Complications	r	0.125
	*p*	0.098
NIHSS 1	r	0.404
	*p*	<0.001
NIHSS1 < 5	r	0.295
	*p*	<0.001
NIHSS2	r	0.734
	*p*	<0.001
NIHSS2 < 5	r	0.565
	*p*	<0.001
mRs	r	0.734
	*p*	<0.001
GCS	r	−0.49
	*p*	<0.001
Anticoagulant/Antiaggregant therapy	r	−0.06
	*p*	0.434
Previous ischemic stroke	r	−0.039
	*p*	0.608
TOAST	r	−0.423
	*p*	<0.001
Vertigo	r	−0.034
	*p*	0.658
Nausea/Vomiting	r	0.085
	*p*	0.261
Diplopia	r	0.146
	*p*	0.055
Instability	r	−0.069
	*p*	0.361
Dysphagia	r	−0.135
	*p*	0.075
IVBSS	r	0.419
	*p*	<0.001
IVBSS > 4	r	0.203
	*p*	0.007

**Table 9:** Prediction of fatal outcome—univariate binary logistic regression.

	OR (95%CI)	*p*-Value
CTP	0.378 (0.162–0.881)	0.024
Gender		
Age	1.041 (1.009–1.075)	0.013
Time from symptoms to examination		
Time longer than 6 h		
Time longer than 9 h		
Hyperdensity	0.229 (0.089–0.588)	0.002
ASPECT score	0.667 (0.445–0.998)	0.049
ASPECT score < 8		
Territory		
Type of blood vessel	4.833 (1.319–17.717)	0.017
Blood vessel: symptomatic/asymptomatic	4.830 (2.191–10.647)	<0.001
Fazekas score		
Positive control NCCT		
Hypertension		
Atrial fibrillation		
Diabetes mellitus		
Blood lipids		
Smoking		
Alcoholism		
Obesity	3.548 (1.135–11.093)	0.029
Therapy		
Complications	0.143 (0.025–0.816)	0.029
NIHSS 1	1.216 (1.130–1.310)	<0.001
NIHSS 2	1.249 (1.162–1.342)	<0.001
NIHSS 1 < 5	0.180 (0.071–0.455)	<0.001
NIHSS 2 < 5		
mRs		
GCS	0.679 (0.580–0.795)	<0.001
Anticoagulant/Antiaggregant therapy		
Previous ischemic stroke		
TOAST	10.563 (3.669–30.408)	<0.001
Vertigo		
Nausea/Vomiting		
Diplopia		
Nystagmus		
Instability		
Dysphagia		
IVBSS	1.230 (1.140–1.328)	<0.001
IVBSS < 4	0.251 (0.084–0.751)	0.013

OR (95%CI)—odds ratio (95% confidence interval).

**Table 10:** Prediction of fatal outcome—multivariate binary logistic regression.

	OR (95%CI)	*p*-Value
CTP	0.245 (0.063–0.952)	0.042
Age		
Hyperdensity		
Blood vessel: symptomatic/asymptomatic		
Obesity		
Complications	2.964 (1.132–7.762)	0.027
NIHSS 1		
GCS		
TOAST	0.522 (0.329–0.830)	0.006
IVBSS	1.217 (1.005–1.473)	0.045
ASPECT score		

OR (95%CI)—odds ratio (95% confidence interval).

**Table 11:** Cumulative number of deaths at different time points according to CTP status.

Died		Time (Day)			
	30	100	500	1000	1500
CTP-positive	30 (25.9%)	31 (26.7%)	33 (28.5%)	34 (29.3%)	34 (29.3%)
CTP-negative	8 (13.6%)	8 (13.6%)	8 (13.6)	8 (13.6%)	8 (13.6%)

## Data Availability

The human data that support the findings of this study are not openly available, due to sensitivity and privacy, and are available from the corresponding author upon request.

## Abbreviations

The following abbreviations are used in this manuscript:

IS	Ischemic stroke
PCIS	Posterior circulation ischemic stroke
TOAST	Trial of Org 10172 in Acute Stroke Treatment
NIHSS.	National Institutes of Health Stroke Scale
IVBSS	Israeli Vertebrobasilar Stroke Scale
NCCT	Non-contrast computed tomography
ASPECTS	Alberta Stroke Program Early CT Score
pc-ASPECTS	Posterior Circulation Alberta Stroke Program Early CT Score
CTP	Computed tomography perfusion
ROI	Region of interest
CTA	Computed tomography angiography
GCS	Glasgow Coma Scale
mRS	Modified Rankin Scale
AIF	Arterial input function
VOF	Venous output function
CBF	Cerebral blood flow
CBV	Cerebral blood volume
MTT	Mean transit time
Tmax	Time to maximum residue function
LAA	Atherosclerosis of large blood vessels
Lacune	Occlusion of small blood vessels
OTH	Stroke of other determined etiology
UND	Stroke of undetermined etiology
SPSS	Statistical Package for the Social Sciences
CAPS	Critical Area Perfusion Score
